# Genotoxicity of Some Metal-Based Antineoplastics, Evaluated by SOS Chromotest and Cytogenetic Analysis

**DOI:** 10.1155/MBD.1996.91

**Published:** 1996

**Authors:** Carmen Socaciu, Loan Pasca, Cristian Silvestru, Ionel Haiduc

**Affiliations:** 1 Department of Biochemistry University of Agricultural Sciences and Veterinary Medicine Cluj-Napoca RO-3400 Romania; 2 Department of Genetics University of Agriculture sciences and Veterinary Medicine Cluj-Napoca Romania; 3 Chemistry Department “Babes-Bolyai” University Cluj-Napoca Romania

## Abstract

The paper reports the screening results of two metal-based antineoplastic drugs with mutagenic potential, such as Romcis (trademark of Cisplatinum, produced in Romania) and diphenylantimony(III) diisopropyldithiophosphate (PADTF). Their effects were compared with those induced by Cyclophosphamide. Two mutagenicity tests, the SOS Chromotest and cytogenetic analysis were applied. The tests were carried out with or without metabolic activation (addition of S_9_-mix), either in E. coli PQ 37 cultures, using four doses (0.3, 3, 30 and 300 pmol compound/assay) for the SOS Chromotest or in leukocyte cultures using 0.3 mM from each compound, for cytogenetics. The dose- response relationships and SOSIP values revealed an indirect mutagenic potential for Cyclophosphamide, amplified by S_9_ mix in bacterial cultures and an antiproliferative, clastogenic effect on lymphocytes. For Romcis and diphenylantimony(III) diisopropyldithiophosphate, a significant positive response by SOS Chromotest was recorded, which correlated with increased frequencies of chromosomal aberrations.


### GENOTOXICITY OF SOME METAL-BASED ANTINEOPLASTICS, EVALUATED BY SOS CHROMOTEST AND CYTOGENETIC ANALYSIS

Carmen Socaciu,*[1] Ioan Pasca,[2] Cristian Silvestru,[3] and Ionel Haiduc[3]

[1] Department of Biochemistry, University of Agricultural Sciences and Veterinary Medicine, RO-3400 Cluj-Napoca, Romania.
[2] Department of Genetics, University of Agricultural Sciences and Veterinary Medicine, Cluj-Napoca
[3] Chemistry Department, "Babes-Bolyai" University, Cluj-Napoca

### ABSTRACT

The paper reports the screening results of two metal-based antineoplastic drugs with mutagenic potential, such as Romcis (trademark of Cisplatinum, produced in Romania) and diphenylantimony(III) diisopropyldithiophosphate (PADTF). Their effects were compared with those induced by Cyclophosphamide. Two mutagenicity tests, the SOS Chromotest and cytogenetic analysis were applied. The tests were carried out with or without metabolic activation (addition of $S_9$-mix), either in E. coli PQ 37 cultures, using four doses (0.3, 3, 30 and 300 pmol compound/assay) for the SOS Chromotest or in leukocyte cultures using 0.3 mM from each compound, for cytogenetics. The dose-response relationships and SOSIP values revealed an indirect mutagenic potential for Cyclophosphamide, amplified by $S_9$ mix in bacterial cultures and an antiproliferative, clastogenic effect on lymphocytes. For Romcis and diphenylantimony(III) diisopropyldithiophosphate, a significant positive response by SOS Chromotest was recorded, which correlated with increased frequencies of chromosomal aberrations.

### INTRODUCTION

The selection of the most appropiate tests for the control of genotoxicity induced by physical or chemical agents, including drugs is still a subject of debate. A unique strategy is not yet established, although a lot of short-term or long-term test batteries have been developed.[1-4]

A simple sequential testing strategy recommends as a first step, *in vitro* tests like Salmonella Assay (Ames test) and cytogenetic analysis on lymphocytes or CHO cells.[3] These tests proved to be good predictive tools for estimating the carcinogenic potential, by the association of a bacterial assay with the chromosomal aberrations test, which detects complementary critical genetic end-points.

A bacterial test, called the SOS Chromotest, was recently described and proved to be a very convenient short-term test for the screening of genotoxic agents.[5,6] This test quantifies the SOS-response of a particular strain, E. coli PQ 37, to DNA-damaging agents by the activation of the structural *lac* Z gene functions (for β-galactosidase synthesis) which is fused with the *sfi*A control gene.[7,8] The β-galactosidase and alkaline phosphatase activities are quantitative markers of genotoxicity, easily determined in a very short time. A validation of this test has been reported for hundreds of chemicals.[6,9] The good correlation obtained with the Salmonella test suggests that the SOS Chromotest is a complementary screening assay in a short-term battery for detecting genotoxins.

The International Commission for Protection against Environmental Mutagens and Carcinogens recommends the use of cytogenetic analysis as a screening test for monitoring mutagenicity.[10] *In vitro* cytogenetics can utilise animal or human lymphocytes[11] for evaluating the chromosomal aberrations as an end-point of mutagenicity.

The experimental data concerning the inhibitory effects of platinum coordination compounds on E. coli division rapidly led to the testing of their potential in cancer chemotherapy.[12,13] Their mechanisms of action, common to other metal coordination compounds are now elucidated[14-16] and some of them are used as cytostatics.[17]

Our interest has been focused on the screening of new chemicals or antineoplastic drugs with mutagenic potential, using the most relevant "in vitro" short-term tests. In this context, we were interested to compare two tests - one performed with a bacterial system and the other one with mammalian cells - for genotoxic potential of some antineoplastics.

Cyclophosphamide, a well known antineoplastic, revealed conflicting results by different genotoxicity tests.[6] The possible use of organometallic compounds as antitumor agents[13,18,19] stimulated our interest to investigate their mutagenic potential. Recently, the genotoxicity of 14 organotin compounds (butyltins, phenyltins and methyltins) established using the SOS Chromotest and the rec-assay tests was reported.[20]

We report here the results obtained using three antineoplastics: Cyclophosphamide, Romcis (Romanian trademark of Cisplatinum) and diphenylantimony(III) diisopropyldithiophosphate, which were comparatively investigated by SOS Chromotest, with or without metabolic activation and by cytogenetic analysis, in blood lymphocytes. Their genotoxic potential is discussed.

### MATERIALS AND METHODS

*Tested compounds*. Two Cyclophosphamide trademarks were tested: the first one Romanian Ciclofosfamida (**CFr**), produced by Drugs Entreprise, Bucharest, Romania and the second one, German Cyclophosphamide (**CFg**) produced by VEB JenaPharm, Jena, Germany. Two metal compounds were comparatively investigated: Romcis (**RC**), produced by the Institute of Chemistry, Timisoara, Romania, and a new organometallic compound possessing antitumor activity, diphenylantimony(III) diisopropyldithiophosphate (abbreviated in the subsequent discussion as **PADTF**).[18,19]

The metal compounds, **RC** and **PADTF** were first dissolved in DMSO at $10^{-3}$M and afterwards three successive dilutions to $10^{-4}$, $10^{-5}$ and $10^{-6}$M were applied. The same concentrations were made for **CFr** and **CFg**, using distilled water as solvent. Considering the dilution factor in the cell suspensions (1:30), the effective final concentrations in bacterial media were $3x10^{-8}$M, $3x10^{-7}$M, $3x10^{-6}$M, $3x10^{-5}$M, respectively and $3x10^{-5}$M in the lymphocyte suspension.

*Bacterial strain, buffers and equipment for SOS Chromotest.* The experiments were performed using the standard tester strain, E. coli PQ37, kindly offered by Prof. M.Hofnung, Institute Pasteur, Paris, together with other chemical reagents needed to perform the SOS Chromotest.

Tris(hydroxymethyl)aminomethane (Tris), sodium dodecylsulphate (SDS) and dimethyl-sulphoxide (DMSO) were Merck products. Glucose-6-phosphate (G6P) and nicotinamide adenine dinucleotide phosphate (NADP) were from Sigma. The buffers, media (**La, L**) and other reagents, including the activation mixture $S_9$ -mix were made as previously described[5] : the liver $S_9$ microsome fraction was prepared from Aroclor 1254-treated rats and the composition of $S_9$-mix was the following : 10 % S9, 61% **L** medium, 25% Tris buffer 0.4 M - pH 7.4, 1.5% NADP 0.1 M, 0.5% G6P 1 M, 2% salt solution ( 1.65 M KCl + 0.4 M $MgCl_2$. $6H_2O$).

*Cultures and testing conditions for the SOS Chromotest.* The assays were performed starting from an overnight culture of E. coli PQ 37 at 37°C, obtained by the dilution of 0.05 ml of frozen culture of tester strain in 5 ml of **La** medium. A volume of 0.5 ml of overnight culture was added to 20 ml **La** medium and the mixture was maintained at 37°C in a gyratory incubator. The cell suspension was checked for its density, which reached a value of $5x10^7$ cells/ml after 2.5 hrs. Then, 5 ml of culture were diluted, either in 5 ml of fresh **L** medium for the assays without activation or in 5 ml of $S_9$-mix, for metabolic activation. Fractions containing 0.6 ml were distributed in disposable stoppered glass test tubes containing 20 ml from each dilution, corresponding to the following doses per assay: 0.3, 3.0, 30 and 300 pmol. The mixtures were incubated with shaking for 2 hrs at 37°C and then each suspension was equally divided into two tubes, the first one for the β-galactosidase (β-Gal) assay and the second one for the alkaline phosphatase (P-ase) assay. The assays were performed concomitantly: for β -galactosidase, 0.3 ml suspension was added to 2.5 ml **B** buffer while for alkaline phosphatase to 2.5 ml **P** buffer. Both mixtures were developed as previously described[8], the absorbances were read at 420 nm ($A_{420}$) against a control (C) containing 0.3 ml **L** medium or $S_9$-mix instead of cell suspensions.[6]

The enzyme activities and the R ratios were calculated from simplified formula[5] :

$$\text{Enzyme units} = 1000 \times A_{420} / t$$ , where t - length of incubation (min)

$$R = \beta\text{-Gal units} / \text{P-ase units}$$

The induction factor (I) represents the ratio between R values registered at different concentrations to the R values registered for control. The SOS-inducing potency (SOSIP) is the slope of the linear region of the dose-response curves and represents the induction factor per nanomole of compound tested in the absence or presence of activation mixture $S_9$ -mix[5].

*Cytogenetic analysis.* Bovine blood lymphocytes were separated and cultured in RPMI-1640 medium containing 15% **FCS**, at a density of 2x $10^6$ cells/ml and supplemented with 20 µg/ml **PHAM**.

All the tested compounds were added to the cell suspension at the begining of 72-h cultures, using for each one the same concentration, $3x10^{-5}$M, corresponding to the highest concentration used in the SOS Chromotest. Control cultures (C) containing the same volume of DMSO and also negative control (NC) cultures, without DMSO, were concomitantly treated. All cultures were harvested after 72 hrs and the chromosome preparations were made according to standard procedures. We analysed 100 Giemsa-stained metaphases per culture and the most important chromatid or chromosome aberrations (breaks, exchanges) were counted and classified.

### RESULTS AND DISCUSSION

*SOS Chromotest : dose-response relationships*

Tables I and II represents the enzyme activities expressed as β-Gal and P-ase units, as well the R and I values determined for all compounds in the absence or in the presence of $S_9$-mix. Using the same doses for each compound, from 0.3 to 300 pmol per assay, different responses were registered for cyclophosphamides (**CFr** and **CFg**) and metal compounds (**RC** and **PADTF**). If the enzyme activities were almost constant for increasing doses of **CF**, a gradual increase of activities was observed for **RC** and **PADTF**.

**Table I.** SOS Chromotest results: enzyme activities, R and I values in the absence of activation ( - $S_9$-mix).

| Compound (pmol/assay) | β-Gal units | P-ase units | R | I |
|---|---|---|---|---|
| **Control** | 2.3 | 20.6 | 0.113 | 1 |
| **CFr/CFg** | | | | |
| 0.3 | 11.1/11.4 | 29.2/28.6 | 0.38/0.39 | 3.4/3.5 |
| 3.0 | 12.1/12.3 | 28.8/27.7 | 0.42/0.44 | 3.7/3.9 |
| 30.0 | 12.2/12.2 | 32.2/30.8 | 0.38/0.39 | 3.3/3.5 |
| 300.0 | 12.2/12.4 | 36.4/32.5 | 0.33/0.38 | 2.9/3.4 |
| **RC** | | | | |
| 0.3 | 11.1 | 20.4 | 0.54 | 4.82 |
| 3.0 | 12.2 | 21.2 | 0.57 | 5.08 |
| 30.0 | 13.1 | 20.8 | 0.63 | 5.56 |
| 300.0 | 18.3 | 22.4 | 0.82 | 7.23 |
| **PADTF** | | | | |
| 0.3 | 11.7 | 28.4 | 0.41 | 3.63 |
| 3.0 | 14.9 | 28.4 | 0.52 | 4.63 |
| 30.0 | 22.1 | 38.6 | 0.57 | 5.06 |
| 300.0 | 42.1 | 46.5 | 0.90 | 8.0 |

In the absence of activation mixture, at the highest dose, the induction factor (I) had values of 2.9 - 3.3 for **CF** and 7.23 or 7.99 for **RC** and **PADTF**, respectively. With metabolic activation, these values increased for all compounds but significantly ($p < 0.005$) only for **RC** and **PADTF**.

The dose-response curves obtained from these data were linear, almost dose-independent for **CF** or had a slight increasing slope for β-Galactosidase (β-Gal) activity beginning with 3 pmol/assay for **RC** and **PADTF**, while the alkaline phosphatase (P-ase) activity was constant. The presence of $S_9$-mix induced in all cases increases of β-Gal activity, slight decreases of P-ase activity and higher induction factors, especially for **RC** and **PADTF**.

**Table II.** SOS Chromotest results: enzyme activities, R and I values in the presence of activation ( + $S_9$-mix).

| Compound (pmol/assay) | β-Gal units | P-ase units | R | I |
|---|---|---|---|---|
| **Control** | 1.1 | 10.3 | 0.11 | 1 |
| **CFr/CFg** | | | | |
| 0.3 | 11.1/11.6 | 20.2/20.6 | 0.55/0.56 | 4.9/5.0 |
| 3.0 | 12.1/12.4 | 20.4/21.2 | 0.59/0.58 | 5.3/5.2 |
| 30.0 | 12.4/12.6 | 21.1/21.8 | 0.59/0.58 | 5.3/5.2 |
| 300.0 | 12.6/12.6 | 21.4/21.8 | 0.59/0.58 | 5.3/5.2 |
| **RC** | | | | |
| 0.3 | 12.3 | 16.2 | 0.76 | 6.8 |
| 3.0 | 14.6 | 17.4 | 0.84 | 7.5 |
| 30.0 | 26.8 | 17.6 | 1.52 | 13.7 |
| 300.0 | 32.2 | 18.2 | 1.77 | 15.9 |
| **PADTF** | | | | |
| 0.3 | 12.1 | 22.4 | 0.53 | 4.8 |
| 3.0 | 26.2 | 26.1 | 1.02 | 9.0 |
| 30.0 | 38.2 | 28.1 | 1.35 | 12.2 |
| 300.0 | 62.1 | 30.4 | 2.03 | 18.3 |

*SOS Chromotest : the induction factors and SOSIP*

Figure 1 represents the induction kinetics for all tested compounds. For both **CF** products the I values were almost independent of the dose and of the addition of $S_9$-mix. For **RC** and **PADTF**, the I values had a linear increasing kinetic, stimulated by the addition of $S_9$-mix. From the slopes of I = f(dose), the SOSIP values were calculated and compared for all tested compounds, in the presence or absence of $S_9$-mix (Table III). These values are larger in the presence of $S_9$-mix than in its absence especially for **RC** and **PADTF** : in the absence of $S_9$-mix SOSIP values were 8 and 14.5 and by $S_9$-mix addition, these values reached at 30.4 and 44.9, respectively. These values reveal for **RC** and **PADTF**

a significant mutagenic potential, amplified by metabolic activation and a slight genotoxicity for both CF products.

**Table III.** SOS-inducing potency (SOSIP) of four antineoplastics tested in the absence or presence of activation mixture.

| Compound | S O S I P (I/nmol) - $S_9$-mix | S O S I P (I/nmol) + $S_9$-mix |
|---|---|---|
| CFr | 0 | 1.1 |
| CFg | 0 | 0.4 |
| RC | 8 | 30.4 |
| PADTF | 14.5 | 44.9 |

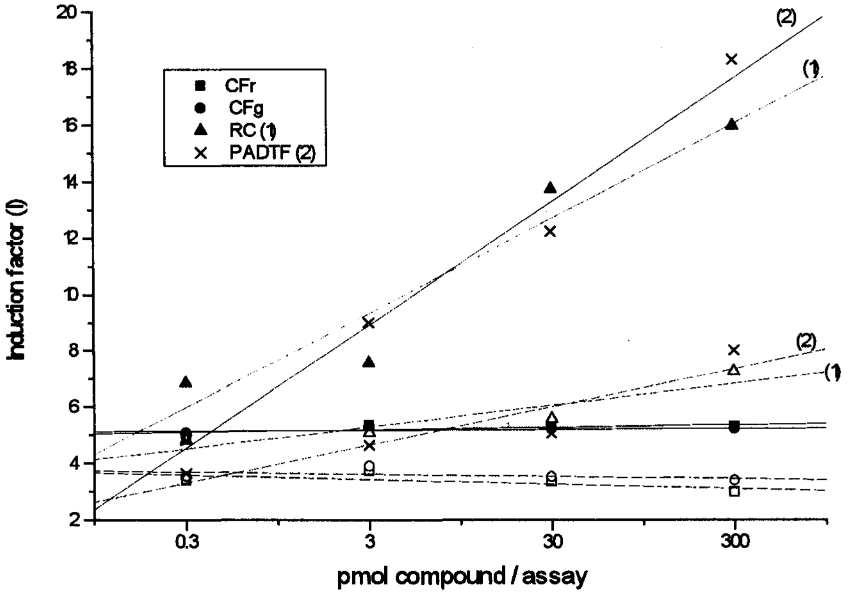

**Figure 1.** Induction kinetics (dose-response slopes) registered for **CFr**, **CFg**, **RC** and **PADTF** treatments with (solid symbols and lines) or without $S_9$ -mix ( open symbols with dashed lines).

*Chromosomal analysis*

Table IV contains the mean values of mitotic index (MI) and the frequencies of chromatid and chromosome aberrations (breaks and exchanges), expressed per 100 metaphases examined. A general decrease of MI for experimentals compared to controls was recorded, especially for **RC** and **PADTF**. A similar observation was made for aberrations, which had higher frequencies for **RC** and **PADTF** treatments, especially at chromatid level (breaks and exchanges). Figure 2 shows two metaphases containing chromatidic breaks and exchanges after the treatment with **RC** and **PADTF**.

**Table IV.** Chromosomal analysis of lymphocytes treated with antineoplastics: mean values of mitotic index (MI) and frequencies of lymphocyte chromatid/chromosomal abberations (expressed per 100 cells scored on slides). Abbreviations : B- breaks; E- exchanges.

| Compound | MI (%) | Chromatid abb. B/E | Chromosome abb. B/E | Total abb. (%) |
|---|---|---|---|---|
| Control | 6.5 | 2/0 | 1/0 | 3 |
| CFr | 4.6 | 1/1 | 1/0 | 3 |
| CFg | 4.5 | 1/2 | 1/0 | 4 |
| RC | 2.5 | 3/2 | 2/1 | 8 |
| PADTF | 1.5 | 3/3 | 2/1 | 9 |

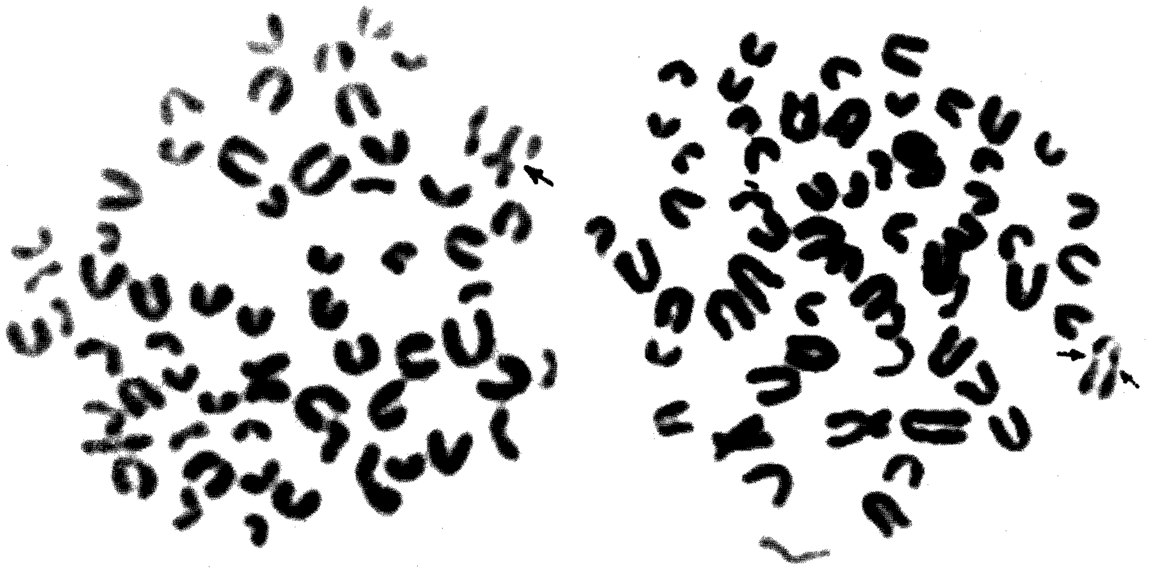

(a)　　(b)

**Figure 2.** Two metaphases showing aberrations ( breaks and exchanges, *see arrows*), identified after the treatment with RC (**a**) and PADTF (**b**), respectively.

The cytogenetic analysis shows similar differences between the effects of **CF** products and **RC** or **PADTF**. The lymphocyte mitotic index was gradually inhibited from **CF** (4.5-4.6) to **RC** (2.5) and **PADTF** (1.5), while the frequency of aberrations was gradually increased in the same order (**PADTF** > **RC** > **CFr** = **CFg**) (Table IV). These results suggest a significant clastogenic potential for **RC** and **PADTF** and a questionable effect for **CF**.

An important aspect for mutagen screening is the analysis of data and legislative guidelines. Different criteria [6, 21] are used for a correct evaluation of SOS Chromotest data but two factors are considered as obligatory for a positive response : an increase of at least 50% for the induction factor and an evident dose-response relationships.

In this respect, we compared our results with cumulative reviews on the mutagenic potential of the tested compounds. For Cyclophosphamide, conflicting responses were reported by SOS Chromotest, Mutatest (Ames test),[4,6] by Micronucleus test[22,23] and carcinogenicity in rodents.[4] In bacterial systems, the SOS-response was negative, the Mutatest was positive while in mammals the response was generally positive[1]. For platinum derivatives the mutagenic potential against E. coli is known since its first evaluation. Positive responses were obtained using also the Salmonella test,[16] the SOS Chromotest[6] and by cytogenetic analysis on human lymphocytes.[4,22,23]

Among antimony-containing derivatives, only antimony sodium tartrate was mutagenic to lymphocytes[4] . Recently[20] , some butyltin oxides and chlorides showed high SOS-inducing factors (3.2 to 5.8). Ghosh et al.[24,25] reported increases in micronuclei and chromosomal aberrations induced by trimethyltin chloride in human lymphocytes. No data about organoantimony compounds were so far reported.

Our results demonstrate similar response for both Cyclophosphamide products, *i.e.* a positive one considering the induction factors but showing weak SOS-inducing potential, with equivocal dose-response relationships. This effect was amplified by metabolic activation, thus confirming the indirect mutagenic potential of Cyclophosphamide. The cytogenetic analysis revealed a moderate antiproliferative and clastogenic effect on lymphocytes, although no quantitative limits for clastogenicity are actually established.

For Romcis and PADTF, intense positive responses were recorded, with significant high values of induction factors, dose-dependence and a greater effect by activation with $S_9$-mix. The dose-response curves had linear increasing slopes which correlate with high SOS-inducing potency. The cytogenetic analysis confirmed their genotoxic potential by increased antiproliferative and chromosomal aberration potency. The magnitude of these effects was greater than for Cyclophosphamides.

Further investigations are in progress, in order to establish the dose-response relationships for a wider concentration range and by complementary mutagenicity tests like the micronucleus test, sister-chromatid exchange. These supplementary investigations could allow a better discrimination among real and false responses and to check the reproducibility of the present results.

### Acknowledgements

The authors are grateful to Dr. Maurice Hofnung and Dr. P.Quillardet (Pasteur Institute, Paris, France) for their kind help, the tester strain E coli PQ 37 and all chemicals needed for performing the SOS-Chromotest. We also thank them for useful discussions regarding the experiments and the results presented in this article.

**References**

1. J. Ashby, *Mutation Res.*, 1983, **115**, 177.
2. J. Ashby, *Mutation Res.*, 1988, **204**, 543.
3. F.H. Sobels, *Mutation Res.*, 1986, **164**, 389.
4. M. Ishidate Jr., M.C. Harnois and T. Sofuni, *Mutation Res.*, 1988, **195**, 151.
5. P. Quillardet and M. Hofnung, *Mutation Res.*, 1985, **147**, 65.
6. P. Quillardet and M. Hofnung, *Mutation Res.*, 1993, **297**, 235.
7. S. Venitt and J.M. Parry (Eds.), *Mutagenicity testing - a practical approach*, IRL Press, Oxford, Washington, 1984.
8. S. Venitt, H. Bartsch, G. Becking, R.P.P. Fuchs, M. Hofnung, C. Malaveille, T. Matsushima, M.R. Rajewsky, M. Roberfroid and H.S. Rosenkranz, in *Long-term and short-term assays for carcinogens: a critical appraisal, IARC Scientific Publ. Lyon*, 1986, **83**, 143.
9. P. Quillardet, C. de Bellecombe and M. Hofnung, *Mutation Res.*, 1985, **147**, 79.
10. A.V. Carrano and A.T. Natarajan, *Mutation Res.*, 1988, **204**, 379.
11. A.K. Sinha, B.B. Gollapudi, V.A. Linscombe and M.L. McClintock, *Mutagenesis*, 1989, **4**, 147.
12. J.J. Roberts, in *Molecular Actions and Targets for Cancer Chemotherapeutic Agents* (A.C. Sartorelli, J.S. Lazo and J.R. Bertino, Eds.), Academic Press, New York, 1981 p. 17.
13. I. Haiduc and C. Silvestru, *Organometallics in Cancer Chemotherapy*, CRC Press, Boca Raton, Florida, Vol.I. Main Group Metal Compounds, 1989; Vol.II. Transition Metal Compounds, 1990.
14. D.J. Beck and R.R. Brubaker, *Mutation Res.*, 1975, **27**, 181.
15. L.A. Loeb and R.A. Zakour, in *Nucleic Acid-Metal Interactions* (T.G. Spiro, Ed.), J.Wiley and Sons, New York, 1980, p. 117.
16. M. Tamaro, S. Venturini, C. Monti-Bragadin, G. Saincich, G. Mestroni and G.Zassinovich, *Chem.- Biol. Interact.*, 1979, **26**, 179.
17. R.K-Y. Zee-Cheng and C.C. Cheng, *Meth. and Find. Expl. Clin. Pharmacol.*, 1988, **10**, 67.
18. C. Silvestru, C. Socaciu, A. Bara and I. Haiduc, *Anticancer Res.*,1990, **10**, 803.
19. C. Socaciu, A. Bara, C. Silvestru and I. Haiduc, *In Vivo*, 1991, **5**, 425.
20. T. Hamasaki, T. Sato, H. Nagase and H. Kito, *Mutation Res.*, 1992, **280**, 195.
21. M. Hofnung and P. Quillardet, *Mutagenesis*, 1986, **1**, 319-330.
22. C. Socaciu, A. Bara and G. Fagarasanu, *Proc.8th Balkan Biochem.Biophys.Days*, 10-14 Sept.1990, Cluj-Napoca (Romania), 1990, p. 314.
23. C. Socaciu, I. Pasca and C. Lisovschi-Chelesanu, *Proc.Symp.18-19 Oct.1990*, Cluj-Napoca (Romania), 1990, **17**, 612.
24. B.B. Ghosh, G. Talukder and A. Sharma, *Mutation Res.*, 1990, **245**, 33.
25. B.B. Ghosh, G. Talukder and A. Sharma, *Mech. Ageing Dev.*, 1991, **57**, 125.

**Received: January 31, 1996 - Accepted: February 26, 1996 - Received in revised camera-ready format: March 21, 1996**

